# Spatiotemporal environmental monitoring of the karst-related Almyros Wetland (Heraklion, Crete, Greece, Eastern Mediterranean)

**DOI:** 10.1007/s10661-023-11571-5

**Published:** 2023-07-15

**Authors:** E. Kokinou, D.E. Zacharioudaki, S. Kokolakis, M. Kotti, D. Chatzidavid, M. Karagiannidou, E. Fanouraki, E. Kontaxakis

**Affiliations:** 1 Department of Agriculture, Hellenic Mediterranean University, Estavromenos, 71410 Heraklion, Greece; 2 Department of Electronic Engineering, Hellenic Mediterranean University, Romanou 3, 73133 Chania, Greece

**Keywords:** Environmental pollution, Physicochemical parameters, Spectral induced polarization, Spectrophotometry, Karstic wetland

## Abstract

Wetlands are of great importance for providing the appropriate conditions for the diversity of species and inhabitants living in these ecosystems and for a wide range of public goods and services (fresh water, air filtering, recreational and tourism opportunities). However, despite their importance, wetlands are continuously degraded due to various human activities at an alarming rate worldwide. In the context of this study and considering the rational management of wetlands and the inevitable impact of climate change, a wetland, spatiotemporal monitoring system is proposed, implemented in the karst wetland of Almyros Stream (Heraklion, Crete, Greece). The implementation of this system is based on robust techniques from the allied fields of chemistry, earth and ecology assisted by GIS techniques. Our results indicate that this Mediterranean karst wetland of great ecological importance is under pressure because of pollution, climate change and land change. This argument is supported by (a) the high concentrations of water photosynthetic pigments and nutrients at certain times of the year, both provided by chemical analysis and partially verified by geophysical monitoring (spectral induced polarization) and (b) the high values of soil magnetic susceptibility at sites close to human infrastructures that is an indicator of heavy metals concentrations.

**Supplementary Information:**

The online version contains supplementary material available at 10.1007/s10661-023-11571-5.

## Introduction

Wetlands are critically important for biodiversity and the people who use their resources or benefit from their many services or functions. Wetland services include protecting and improving water quality, providing habitats for organisms, storing floodwaters and maintaining surface water flow during dry periods. Yet they are disappearing worldwide (Baker, 2006; Finlayson, 2018; Humphreys, 2000; Mediterranean Wetlands Observatory, 2014). In the twentieth century, almost 50% of Mediterranean wetlands disappeared. According to the Mediterranean Wetlands Observatory (2014), the Mediterranean natural wetlands area declined by 10% between 1975 and 2005, representing a total loss of 1248 km^2^ for the 214 sites considered. Among wetlands, wet meadows and marshes are the most affected habitats corresponding to 10% and 43% loss, respectively).

Karst wetlands belong to the threatened wetland types. Less than l0% of the world has distinct karst landscapes, but globally, karst wetlands host the majority of belowground biodiversity (Humphreys, 2000). The term karst refers to a distinctive landform formed mainly by rock dissolution by natural water; hence, the term “landscape of soluble rock” is sometimes used. Because of their solubility, karst is most pronounced in carbonate rocks (limestone and dolomite) and evaporites such as gypsum. Such areas are characterised by sinking streams, caves, closed depressions, rippled rocks and large springs. The integrity of such landscapes depends on the maintenance of the natural hydrological system, and they are potentially very fragile, comparable in this respect to deserts or coastal margins (Humphreys, 2000). Furthermore, karst wetlands, listed as a wetland type in the Ramsar Convention on Wetlands (Matthews, 1993), are a type of subsurface wetland system with or without a surface water component, usually associated with caves or other underground cavities. The components of a karst system—rock, water, soils, flora, fauna, energy and gases—are closely interconnected, and changes to any of these components can affect the entire system, including associated wetlands (Baker, 2006; https://rmi-data.sprep.org/system/files/RMI%20Ramsar%20Sites_appendix7.pdf).

Like most wetlands, karst wetlands offer economical, cultural and conservation values. Because karst wetlands are inherently dependent on the relationships between land, water, soil and vegetation, many threats can affect these systems’ complex biogeochemical and biogeophysical cycles. These threats include human (chemical) pollution, climate change, hydrological encroachment (physical) and inappropriate recreation or human use (Baker, 2006). In addition, karst wetlands have a low capacity to recover from change. This is because pollutants that enter a karst wetland can remain in the karst system for hundreds or even thousands of years. It is, therefore, important to adequately mark tipping points for managing threats.

**Fig. 1:**
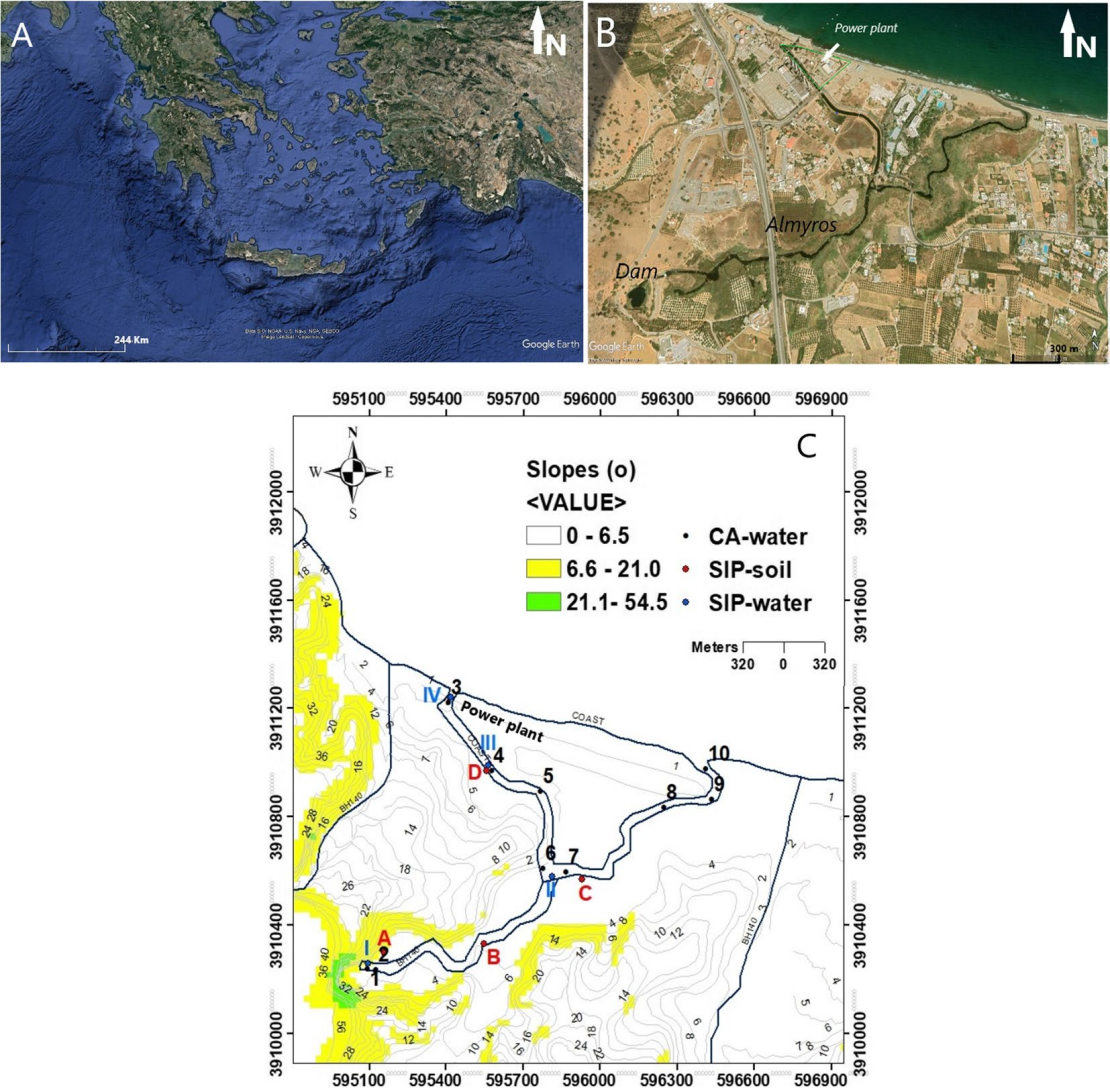
Almyros Wetland. **A** Google Earth map showing the location (see red arrow) of the study area in the central part of Crete (Greece), Eastern Mediterranean Sea, **B** Google Earth satellite map showing details of the study area, a green line indicates the power plant, **C** geomorphological features created from digital 2 m elevation data. The coloured dots show the locations of the soil and water samples for the year 2022

There is a great need to spatially and temporally monitor and further quantify the environmental quality in a karst wetland to raise awareness among policymakers and the public about the environmental degradation of these exceptional habitats (Kokinou et al., 2013; Kotti et al., 2018; Zacharioudaki et al., 2021). Against this background and considering how vulnerable the karst systems are to climate change and human pollution, a new approach is proposed to monitor and quantify the environmental status of karst wetlands. This approach is based on geophysical field and laboratory measurements supported by chemical analyses and integrated with geographic information systems (GIS). It is applied in the fragile karst system of the Almyros (or Almiros) wetland in Heraklion, Crete (Greece, Eastern Mediterranean) (Fig. 1). The objectives of the present work are (a) to demonstrate the effective use of the geophysical, chemical and ecological methods and to interpret their results qualitatively and quantitatively, (b) to use the spatio-temporal analysis as a valuable step in environmental modelling and (c) to establish a monitoring system based on biogeophysical and chemical properties.

This monitoring system will help identify threats, detect tipping points and protect against environmental degradation. The Almyros Wetland is ecologically protected either by European Union protection status or by national legislation. The Almiros Stream, located in the municipality of Malevizi in Crete, Greece, has been recognised as a Wildlife Refuge (IUCN category IV). According to previous studies (Ministry of Environment and Energy - Department of Energy, 2017), the primary uses of the area are wildlife conservation, tourism, agriculture, public services and urban activities. The wildlife reserve represents about 44.82% of the study area and belongs to the state, while 26.08% of the area is used for tourism and public services, such as hotels, shops, a transport network, an electric power plant and a desalination plant. 19.26% of the study area is used for private agriculture and livestock, and 9.84% is an urban area (privately owned). However, due to its coastal location, this wetland is under constant pressure from various factors (tourism, agricultural and industrial activities, domestic and public use and climate change). As far as we know, no measures have yet been taken for this area to monitor its response to environmental degradation.

## The environment of Almyros Wetland

**Fig. 2:**
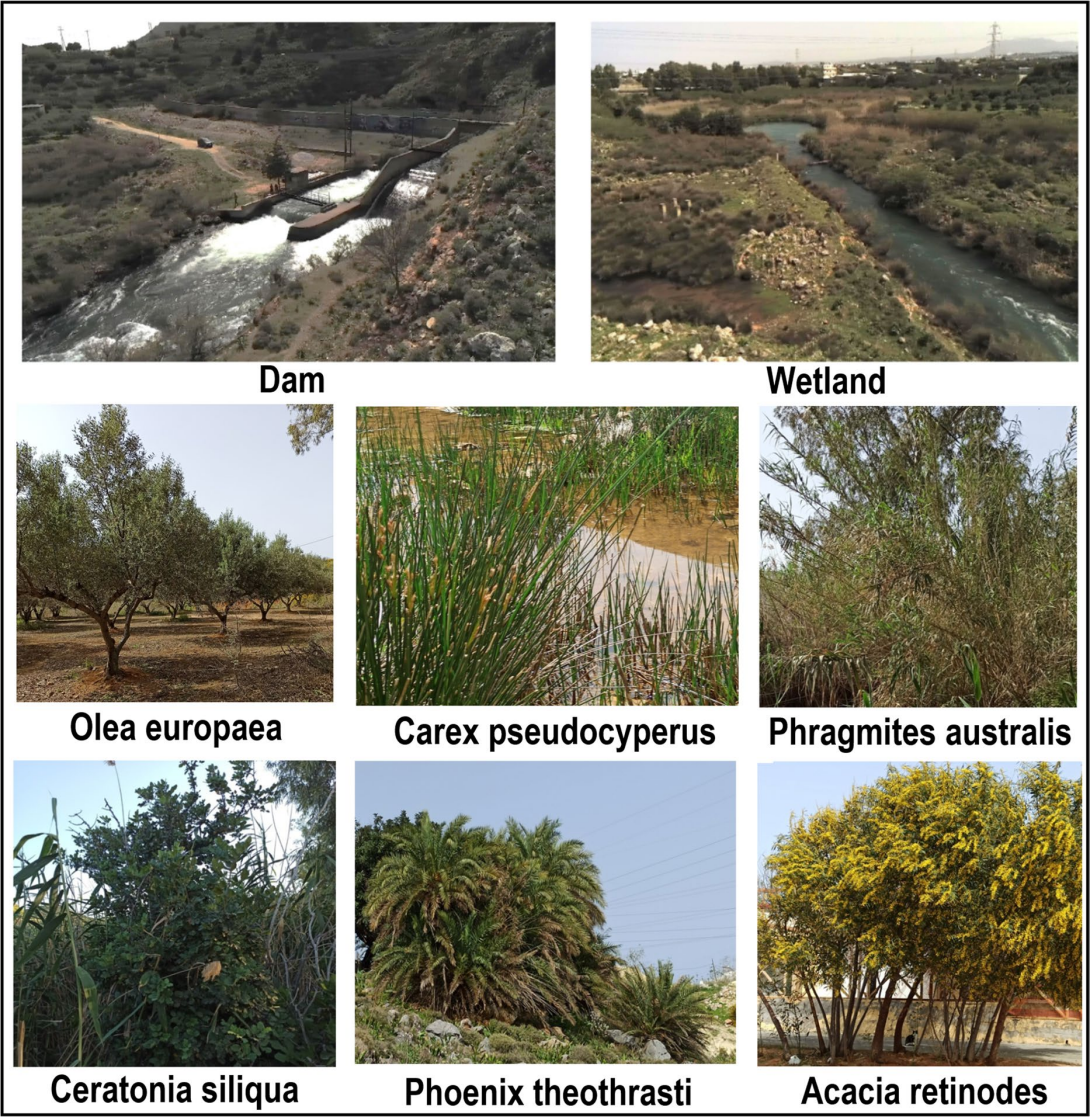
The natural environment of Almyros Wetland

The Almyros Wetland is in the western part of the Heraklion basin in Crete, Greece (Fig. 1A–C). The name “Almyros” refers to the high salt content of the stream in this wetland compared to the water of other rivers in Crete. The Almyros Stream is 1.8 km long and 5–20 m wide (Fig. 2), flows continuously and has an average annual discharge of 235 × 10^6^ m^3^, sufficient to meet the water needs of the greater Heraklion area. The Central Council for Environmental Licencing permitted in 2022 to use small amounts of water (3000 m^3^ per day).

The catchment area of the Almyros Stream is about 300 km^2^ (Afrib et al., 2007) or 500 km^2^ (Lambrakis et al., 2000). Pre-Neogene and Neogene geological units underlain the hydrological basin of Almyros (Monopolis et al., 2005). The dominant geological basement of the area corresponds to carbonate rocks, the so-called semi-autochthon, the permeable unit of “Plattenkalk” of Triassic—Eocene age. The Almyros Wetland includes a sandy beach zone associated with a low relief dune field in front of an alluvial (Holocene in age) plain composed of alluvial, fluvial and marly deposits; the latter is an agricultural area with vineyards, olive trees and pastureland. A desalination plant has been operating in the Almyros Wetland for the last few years to treat the brackish water. There is also a power plant downstream, for the cooling of which an artificial canal 800 m long and 5–10 m wide has been constructed.

The Almyros Wetland is a karst system with a karst spring upstream and a dam downstream where the stream flow is visible (Alexakis & Tsakiris, 2010; Tsakiris & Alexakis, 2014). The spring is located at the bottom of a lake with a diameter of 70–90 m and a depth of up to 20 m. The flow rate of the spring ranges from 4 m^3^/s (hot seasons) to 20 m^3^/s (Giannarou, 2009) and reaches up to 70–80 m^3^/s (cold seasons) (Maramathas et al., 2003).

Potential sources of pollution in the study include public and private infrastructure (transport networks, power plants and power lines, desalination plants, factories, hotels, etc.), agricultural activities, livestock, domestic waste disposal and inert material dropping (https://www.ygrotopio.gr/general/report.php?code=KRI111&lang=el).

The area’s fauna is characterised by various plant species, including trees, perennials and annuals (Table 1, Appendix I). Trees present in the area include species such as *Eucalyptus sp*., *Olea spp*., *Ceratonia siliqua*, *Tamarix spp*. and *Phoenix theophrasti*. *Ceratonia siliqua* is a dioecious tree with economic and cultural importance in the Mediterranean region since ancient times (Viruel et al., 2019). *Tamarix* species are native to many coastal areas of Greece and the Mediterranean near beaches, riverbanks and brackish streams. It tolerates drought and salinity due to its ability to absorb and bind salts (Ohrtman & Lair, 2013). *Phoenix theophrasti*, known as the Cretan palm, is a palm native to the eastern Mediterranean with a small distribution in southern Greece, mainly in Crete and the Peloponnese (Rivera et al., 2019).

In addition to trees, there are various perennial and annual plants in the area. Some examples of perennial plants are *Euphorbia characias*, *Urtica dioica*, *Nicotiana glauca*, *Drimia maritima* and *Phragmites australis*, the most characteristic and dominant plant of the area. *Euphorbia characias* is one of the most common plants in Mediterranean habitats and has been known for its medicinal properties since ancient times (Appendino et al., 2000). *Urtica dioica* is a dioecious herbaceous perennial, an aggressive weed commonly found in wet soils and attributed with medicinal and antioxidant properties (Jan et al., 2017). *Nicotiana glauca* is a non-native, highly invasive alien plant species that grows in many Mediterranean ecosystems (Hulme et al., 2008). *Drimia maritima* is endemic to the Mediterranean and is considered an indicator of overgrazing (Kaltsas et al., 2019).

Annual herbs such as *Cota tinctoria*, *Matricaria chamomilla* and *Salicornia europaea* are also found in the area. *Cota tinctoria* (golden marguerite, yellow chamomile) is an annual plant used to produce dyes in the food industry, also known as a herbal tea for human health (Bahadori et al., 2021). *Matricaria chamomilla* is an annual medicinal plant found in the Mediterranean region. It is considered a medicinal plant due to its extensive therapeutic uses (Chauhan et al., 2022). *Salicornia europaea* seems well adapted to the Almyros Wetland as it is one of the most salt-tolerant plant species, tolerating highly saline water (Lv et al., 2012).

In summary, the Almyros Wetland is characterised by various plant species, including trees, perennials and annuals. These species contribute to the overall health and diversity of the ecosystem, provide food, fresh water, air filtering and shelter for wildlife, add colour and beauty to the landscape, and have a significant contribution to public health (Bahadori et al., 2021; Jan et al., 2017; Ohrtman & Lair, 2013; Rivera et al., 2019).

## Methodology

### Water and soil sampling and preparation

From January to December 2022, water samples for chemical analyses were collected monthly from ten stations (Fig. 1, identified by numbers 1–10). In this work, we also considered conductivity values at stations 1, 3 and 7, measured in 2016 and presented in our previous work by Kotti et al. (2018). All samples were taken from a depth of 10 cm below the water surface and filled into dark 1 L PTFE bottles. The bottles were pre-washed with tap water, deionised water, 5% HCl solution, tap water and finally, deionised water. Most samples were analysed immediately upon arrival at the laboratory or stored at 4 °C to be analysed within 3 days.

In addition, geophysical analyses using spectrally induced polarization (SIP) were carried out for both water and soil samples. Water samples were collected at the end of May 2022 at four stations (Fig. 1, indicated by I-IV) from 10 cm depth below the water surface in plastic bottles (2L) pre-rinsed with tap water. Soil samples were collected in April 2022 at four stations along the Almyros Stream (Fig. 1, indicated by A–D). Four incisions were made in the soil surface to collect the samples, forming a square; then, a shovel was used to collect a cube of undisturbed soil. Finally, reprocessed magnetic susceptibility analyses carried out about 10 years ago (Kokinou et al., 2013) were included in this work, as one of the aims of this study was to establish spatio-temporal relationships between elevated concentrations of anthropogenic particles and known sources of pollution in the Almyros Wetland. 109 topsoil samples from a depth of about 15 cm below the surface were analysed for magnetic susceptibility and thermomagnetism. The soil samples were mixed in plastic containers, air-dried, crushed and sieved to retain the fractions smaller than 2 mm and to reduce the distorting effect of air, water and pebbles.

### Chemical analyses

All chemical reagents used to analyse total hardness, chloride, nutrients and pigments were of analytical grade, purchased from Sigma-Aldrich, unless something else was stated. The determination of total hardness has been implemented using Ethylenediaminetetracetic acid (EDTA) and Eriochrome Black T (EBT) tablets (Merck). Chloride was determined by silver nitrate (AgNO_3_) and potassium chromate (K_2_CrO_4_), while ammonium by Nessler reagent (labkem). For the determination of phosphate, ammonium molybdate ((NH_4_)_6_Mo_7_O_24_. 4H_2_O), concentrated sulfuric acid (Honeywell) and ascorbic acid (labkem) were used. Furthermore, the determination of silicate was done using ammonium molybdate ((NH_4_)_6_Mo_7_O_24_. 4H_2_O), concentrated sulfuric acid (Honeywell), ascorbic acid (labkem) and oxalic acid (labkem). Αcetone (Honeywell) was used for the pigments. Standard solutions of nitrate, ammonium, orthophosphate and silicate were prepared by diluting 100 mg/L stock solutions using the appropriate amounts of KNO_3_, NH_4_Cl, K_2_HPO_4_, SiO_2_. The spectrophotometric measurements were performed by a Hitachi U-2001 dual-beam spectrophotometer.

#### Physicochemical parameters

pH, electrical conductivity (EC) and salinity (S) were measured with a multiparameter probe (Sension 156 Hach). The probe was calibrated for pH with buffers 7.00 (±0.02) and 4.00 (±0.02) and for EC with 1000 (±10) μS/cm NaCl solution (491 ± 2.5 mg/L) purchased by Hach. Total hardness and chloride were measured by titration with EDTA and AgNO_3_ (Mohr method), respectively, according to standard methods of analysis (APHA, 2005).

#### Nutrients

Analysis of nutrients and silicate was performed spectrophotometrically (APHA, 2005). Nitrate (as N) was measured by subtracting the absorbance at 275 nm from the absorbance at 220 nm (LOD = 0.020 mg/L). Ammonium (as N) was measured by the Nessler method, requiring the sample to be filtered before measurement (LOD = 0.005 mg/L). Absorbance was measured at 400 nm. Orthophosphate (as P) was measured using the molybdenum blue method by recording the absorbance at 880 nm (LOD = 0.003 mg/L). Silicate (as Si) was also measured spectrophotometrically using the molybdate method by recording the absorbance at 880 nm. Oxalic acid was added to mask the interference of orthophosphate (LOD = 0.007 mg/L).

#### Photosynthetic pigments

The photosynthetic pigments chlorophyll-a (chl a), chlorophyll-b (chl b), chlorophyll-c (chl c) and carotenoids were determined using the spectrophotometric trichromatic method (APHA, 2005). Samples were filtered through 0.45 μm Millipore filters under vacuum, and the pigments were extracted from the filters with acetone 90%. The spectrophotometric determination was carried out at specific wavelengths (480 nm, 510 nm, 630 nm, 647 nm, 664 nm, 750 nm).

### Geophysical analyses

The PSIP is a geophysical instrument for laboratory and in-situ measurements of near-surface spectrally induced polarization (SIP), conventional resistivity, time-domain induced polarization and self-potential. The method of induced polarization (IP) is not new. It was proposed by Conrad Schlumberger about 100 years ago. Two types of IP methods are used to collect data: spectral-induced polarization (SIP) and time-domain-induced polarization (TDIP). In recent decades, SIP has been widely used for hydrogeological and environmental studies to investigate the flow and transport properties of rocks, fluid content/chemistry and biogeochemical state (Atekwana & Slater, 2009; Kemna et al., 2012; Kirmizakis et al., 2020; Revil et al., 2012). The SIP technique estimates the real and imaginary components of complex conductivity by measuring the phase shift and magnitude of conductivity of an injected current, typically over a wide range of frequencies. The real component corresponds to energy loss (conductivity), and the imaginary component to energy storage (polarization) (Binley & Kemna, 2005; Kemna et al., 2012; Kirmizakis et al., 2020; Slater & Lesmes, 2002). In the present work, we used the portable SIP field/laboratory instrument to determine the SIP response of (a) Almyros water, (b) tap water influence on soil samples from the Almyros Wetland and (c) oil-contaminated soils from the same area.

The samples were placed in tubes (test columns) equipped with two current electrodes at the top and bottom of the tube and the potential electrodes in the middle of the column body. The unit is connected to a monitor via its VGA port. After the device and the test tubes were prepared, the soil samples were crushed, homogenised, and then added to the test tubes with tap water. All measurements were carried out in the frequency range of 0–1000 Hz.

The magnetic susceptibility method was also used to check the study area for high concentrations of heavy metals. In environmental magnetism (Thompson & Oldfield, 1986), the most used magnetic parameter is magnetic susceptibility (χ), which is the ratio of the induced magnetisation of a sample in the presence of a weak magnetic field to the applied field itself (Thompson & Oldfield, 1986). In the present study, the magnetic susceptibility of all samples was measured using the dual-frequency version of the MS2B sensor (Bartington Instruments). Accurate measurements of the mass susceptibility were obtained at two frequencies (f_low_ = 0.43KHz and f_high_ = 4.3KHz). Furthermore, the frequency-dependent susceptibility (χ_FD_%) was calculated using the formula: χ_FD_ (%) = [(χ_LF_ - χ_HF_ )/χ_LF_]x100, where χ_LF_ is the susceptibility measured at low frequency, and χ_HF_ is the susceptibility measured at high frequency.

Kokinou et al. (2013) conducted a geophysical survey in the study area using magnetic susceptibility and thermomagnetism (Hansen et al., 1981; Petrovsky & Ellwood, 1999; Thompson & Oldfield, 1986) to identify sites with contaminated topsoil (Bityukova et al., 1999; Boyko et al., 2004; Petrovsky et al., 2001; Scholger, 1998) regarding heavy metals (Goluchowska, 2001; Kokinou, 2015; Sarris et al., 2009; Strzyszcz et al., 1996). The geophysical data were interpreted considering the area’s topography, geology, urban network and blowing winds. In this work, the data from Kokinou et al. (2013) have been reprocessed to incorporate the earlier information on topsoil contamination into the current analyses. From a geological point of view, most of the samples collected are from Pliocene to Pleistocene and alluvial sediments (Kokinou et al., 2013). A few samples are from the westernmost area covered by the Phyllites-Quartzites unit. Loci with high magnetic susceptibility values within the study area gave rise to thermomagnetic analysis of the selected samples (Kokinou et al., 2013). The thermomagnetic analyses revealed the presence of magnetite as the most important magnetic mineral in the samples and the contribution of haematite (Thompson & Oldfield, 1986).

### Geographic Information System (GIS) and statistical processing

Digitisation techniques and GIS were used to plot the spatial distribution of the sampling stations on the topographic data (contour lines and slopes). The topographic maps of the area (scale 1:5000) were enriched with a generalisation of selected geological formations of the region available on geological maps of the Institute of Geological and Mineral Exploration (IGME) at scale 1:50000. Fault lines, the Almyros Stream and major and minor roads were also digitised and integrated into the project’s Geographic Information System. The cell size of the digital elevation model is 2 m. The inverse distance weighting method was chosen for the data.

Statistical processing using Microsoft Excel took place to investigate the correlations among the physicochemical parameters, nutrients and pigments. Data have been grouped based on salinity in the dry period group (April–October) and wet period group (November–March). The r-squared value was used for correlation, which returns the square of the Pearson product moment correlation coefficient through data points in known y’s and x’s. The r-squared value can be interpreted as the proportion of the variance in y attributable to the variance in x.

## Results

### Water quality characterisation

#### Physicochemical measurements

**Fig. 3:**
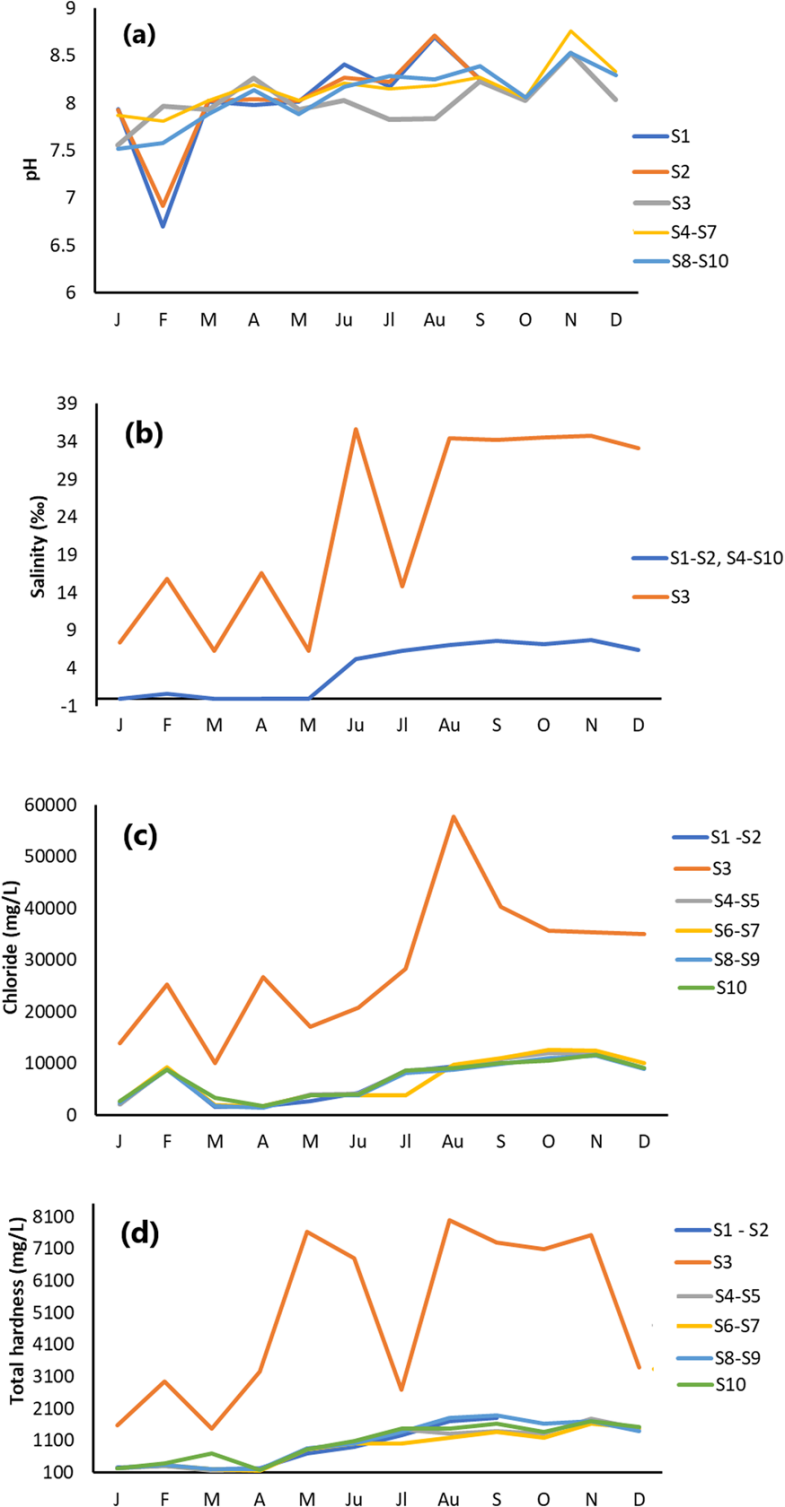
Monthly measurements of physicochemical parameters from January to December 2022 at the ten sampling stations (location in Fig. 1): **a** pH, **b** salinity, with values of salinity at stations 1, 2, 4, 5, 6, 7, 8, 9 and 10 (yellow line) matching for January, February, April and from June to December, **c** chloride, with values (yellow line) almost matching for all months except July and **d** total hardness of water samples, with values (yellow line) matching for January, February, April, May, June, November and December, except for station 3

Figure 3 shows the monthly and spatial fluctuations of the physicochemical parameters. The pH values varied between 6.70 and 8.76 in the study area, with a mean of 8.08 (Table 2). In February, the pH values at stations 1 (pH = 6.7) and 2 (pH = 6.92) were extremely low compared to the other stations, which had similar values (Fig. 3a), with pH ranging between 7.52 and 8.76. At station 3, a decrease in pH was observed in June, July and August.

**Table 2:** Minimum, maximum and mean values of all the measured parameters during the sampling campaign. Zero values indicate that the values were below the limit of detection. The name of the station is in parentheses

Parameter	minimum	maximum	mean
Physicochemical			
pH	6.70 (S1)	8.76 (S4)	8.08
EC (mS/cm)	0.47 (S10)	59.50 (S3)	9.79
Salinity (‰)	0.00	35.60 (S3)	5.58
Chloride (mg/L)	1393 (S8)	57680 (S3)	8739
Total hardness(mg/L)	180 (S10)	8000 (S3)	1342
Nutrients			
N-NH_4_ (mg/L)	0.00 (all)	1.97 (S3)	0.22
N-NO_3_ (mg/L)	0.01 (S3)	1.68 (S7)	0.30
P-PO_4_ (mg/L)	0.00 (all)	0.13 (all)	0.04
Si-silicate (mg/L)	0.00	41.30 (S1)	7.50
Pigments			
Chl a (μg/L)	0.0	94.3 (S5)	11.5
Chl b (μg/L)	0.0	137.2 (S5)	20.4
Chl c (μg/L)	0.0	307.1 (S10)	20.3
Carotenoids (μg/L)	0.0	65.5 (S5)	8.3

Salinity was low in the first 5 months at all sampling sites (between 0 and 2.5‰), except for station 3 (between 6.3 and 35.6‰) and increased significantly from June to the end of the sampling period at all stations (Fig. 3b). In general, salinity values did not show significant spatial variations, except for station 3.

Chlorides varied between 1393 and 57680 mg/L (Fig. 3c, Table 2). In addition, station 3 had higher chloride concentrations compared to the other stations ranging from 10065 to 57680 mg/L, while the highest value was measured at station 3 in August (57680 mg/L) (Fig. 3c). A decrease in chloride was observed at all stations in March and May, while an increase was recorded at all stations in February.

Total hardness varied between 180 and 8000 mg/L (Fig. 3d, Table 2), showing the same behaviour as chloride. It was similar over the year at all stations except for station 3 (Fig. 3d). Low total hardness values were recorded in the first 4 months (from 150 to 380 mg/L) and higher values from May to December, ranging from 700 to 1820 mg/L. Hardness values at station 3 were extremely high compared to the other stations, ranging from 1480 to 8000 mg/L. A significant decrease in hardness was observed only at station 3 in July, while the values at the same station were very high in the other dry months. Station 3 is located directly behind the power plant and faces the sea (Fig. 1C). The Almyros water is used to cool the machinery of the power plant.

**Fig. 4:**
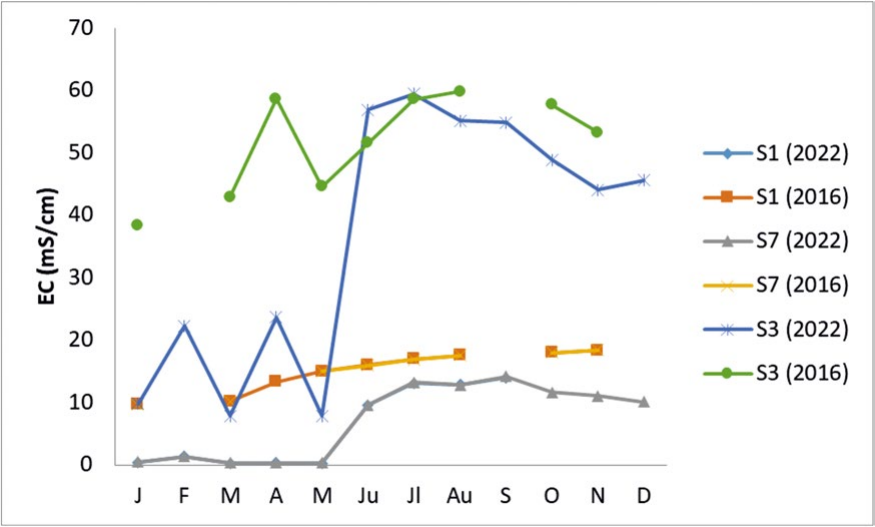
Spatiotemporal electrical conductivity (EC) distribution in Almyros Wetland for 2016 and 2022. S1, S7 and S3 correspond to the stations indicated in Fig. 1

Figure 4 compares the 2016 and 2022 conductivity values at the same sampling points (stations 1 and 7). The conductivity fluctuated both in the same year and the different years (Fig. 4). In particular, the conductivity in 2016 revealed high values that increased from January to the end of the year. The conductivity in 2022 showed low values from January to May. Moreover, a significant increase in conductivity was observed in June 2022, which is close to the values measured in June 2016 (Fig. 4). The same behaviour is observed for station 3. Low conductivity values were measured from January to May 2022, followed by an increase in the next period, reaching the values of June 2016 (Fig. 4).

**Fig. 5:**
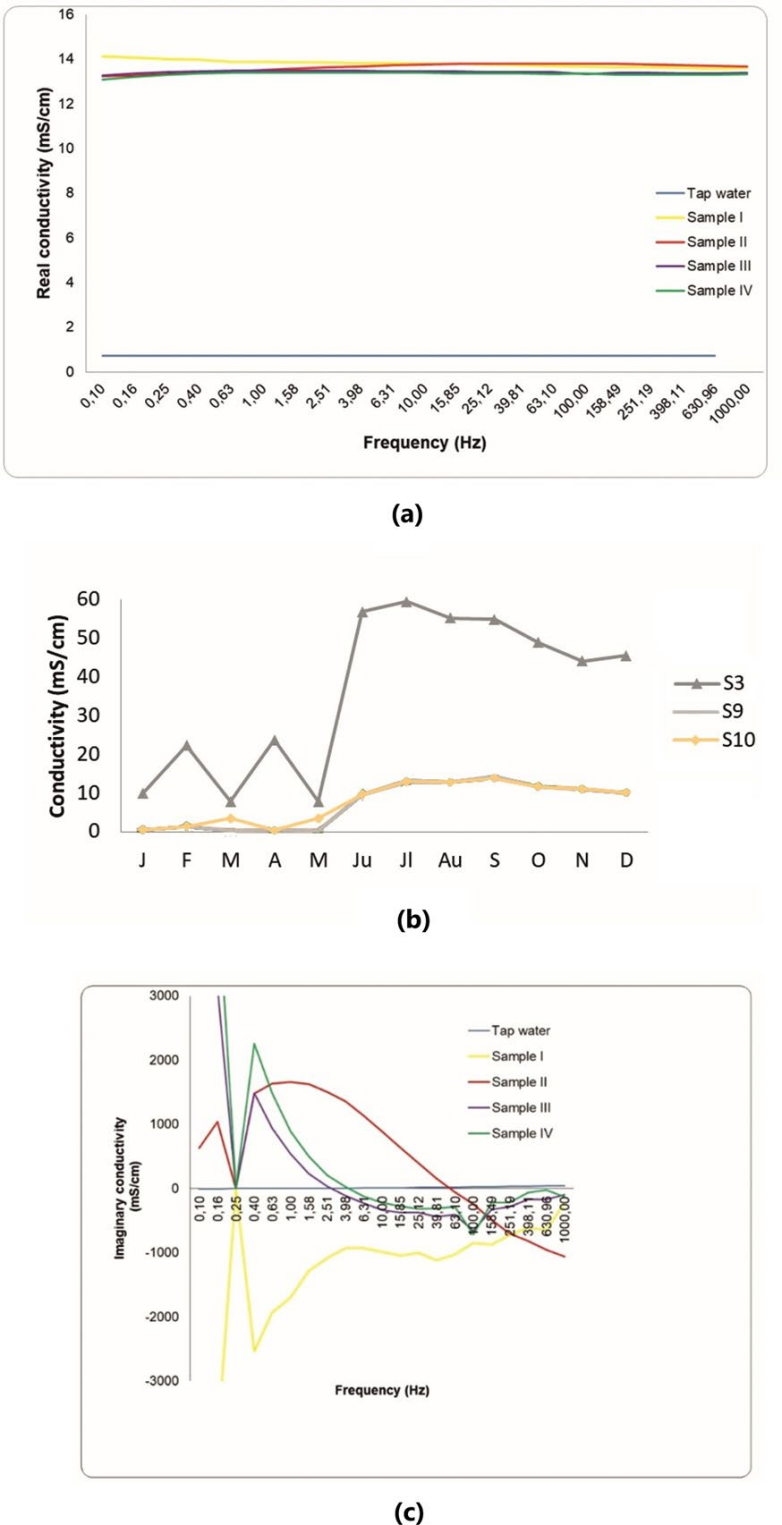
Electrical conductivity of Almyros water: **a** real conductivity (SIP) versus frequency based on geophysical measurements (spectral induced polarization) corresponding to May 2022 at the four sampling stations (location in Fig. 1), **b** spatiotemporal distribution based on chemical analyses (multiparameter probe, Hach) for the period January–December 2022 at the ten sampling stations (location in Fig. 1C), **c** imaginary conductivity (SIP) versus frequency

The SIP response of Almyros water corresponding to the end of May 2022 is shown in Fig. 5a, c while Fig. 5b presents the conductivity detrmined by chemical analysis. The real conductivity (SIP) of the four samples (I, II, III, IV) along the stream ranged from 13.24 to 14.11 mS/cm in the frequency range 0–1000 Hz, while the real conductivity (SIP) of the tap water was 0.72 mS/cm (Fig. 5a). The water conductivity, determined by chemical analysis, varied from 0.472 to 59.5 mS/cm in 2022 (Table 2, Fig. 5b). The real conductivity values measured with the SIP method (Fig. 5a) are close to those of the chemical analysis (Fig. 5b), except for station 3. The imaginary conductivity (SIP) presents large fluctuations in the frequency range 0–1000 Hz and tends to decrease at high frequencies (Fig. 5c).

#### Nutrients concentrations

**Fig. 6:**
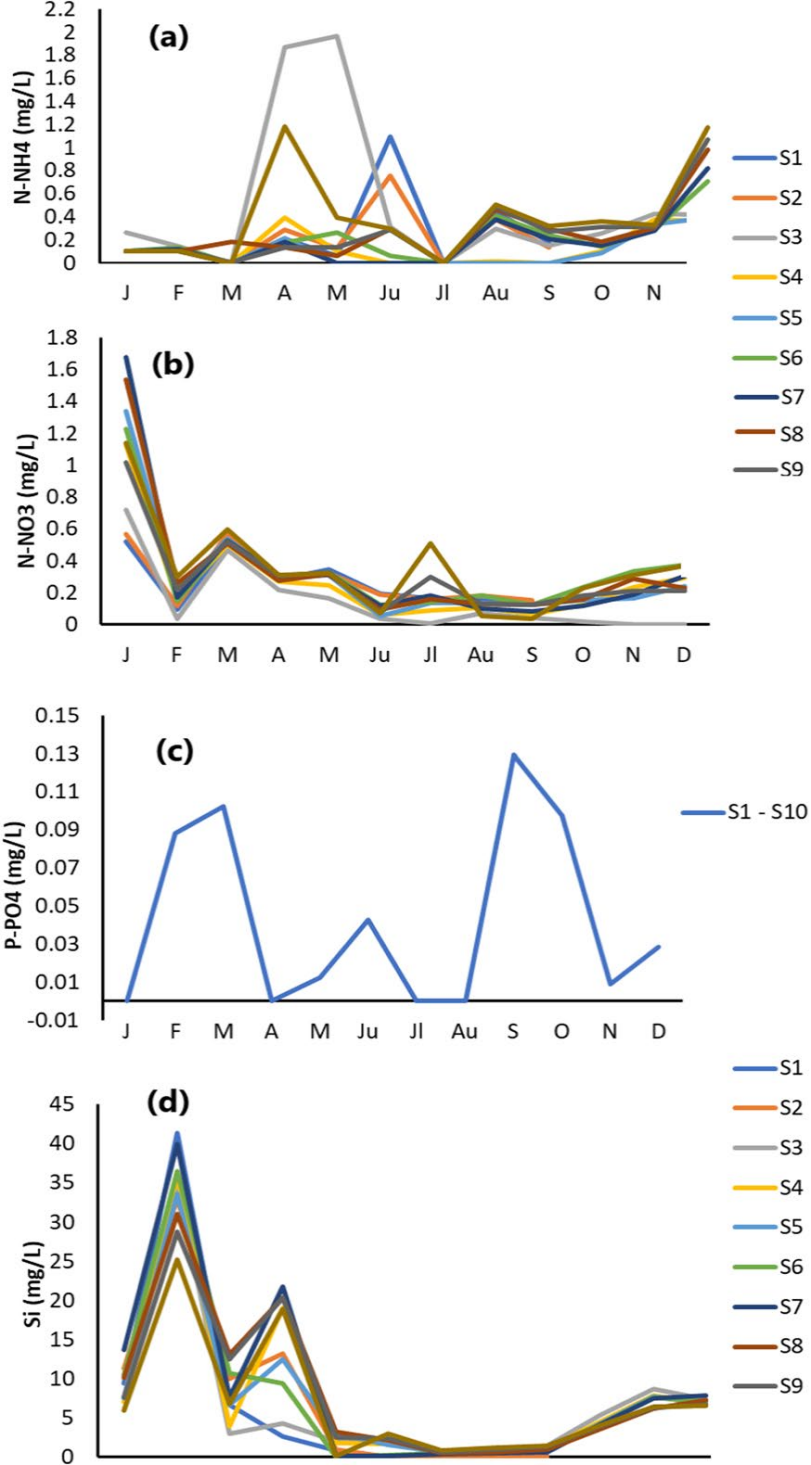
Monthly measurements of nutrients in Almyros water: **a** N-NH_4_, **b** N-NO_3_, **c** P-PO_4_ where the values (yellow line) are similar at all stations, **d** Si where the values (yellow line) are similar at all stations for July, August, September, December

Figure 6 shows the monthly and spatial variations in nutrient concentrations. Ammonium concentrations show spatial and monthly variations ranging between 0 and 1.97 mg/L (Table 2). Specifically, ammonium was high in April, June, August and November, showing the highest values at stations 3 (values up to 1.97 mg/L) and 10 (values up to 1.184 mg/L).

Phosphate, nitrate and silicate did not show spatial but only monthly fluctuations (Fig. 6b, c, d). Nitrate was high in January (from 0.52 to 1.68 mg/L) and March (from 0.47 to 0.57 mg/L) and low in July (from 0.04 to 0.19 mg/L) (Fig. 6b, c, d).

Phosphate levels were high in February (0.088 mg/L), March (0.102 mg/L), July (0.042 mg/L), September (0.129 mg/L) and October (0.097 mg/L), while levels were below the detection limit in the other months (Fig. 6c).

Silicon levels were high in February (between 25.15 and 41.33 mg/L) and April (between 2.64 and 21.74 mg/L), while they were lower in summer (Fig. 6d). In ocean surface waters, silicon concentrations are 30 μg/L, while in rivers they are 4 mg/L (Istvánovics, 2009).

#### Concentrations of photosynthetic pigments

**Fig. 7:**
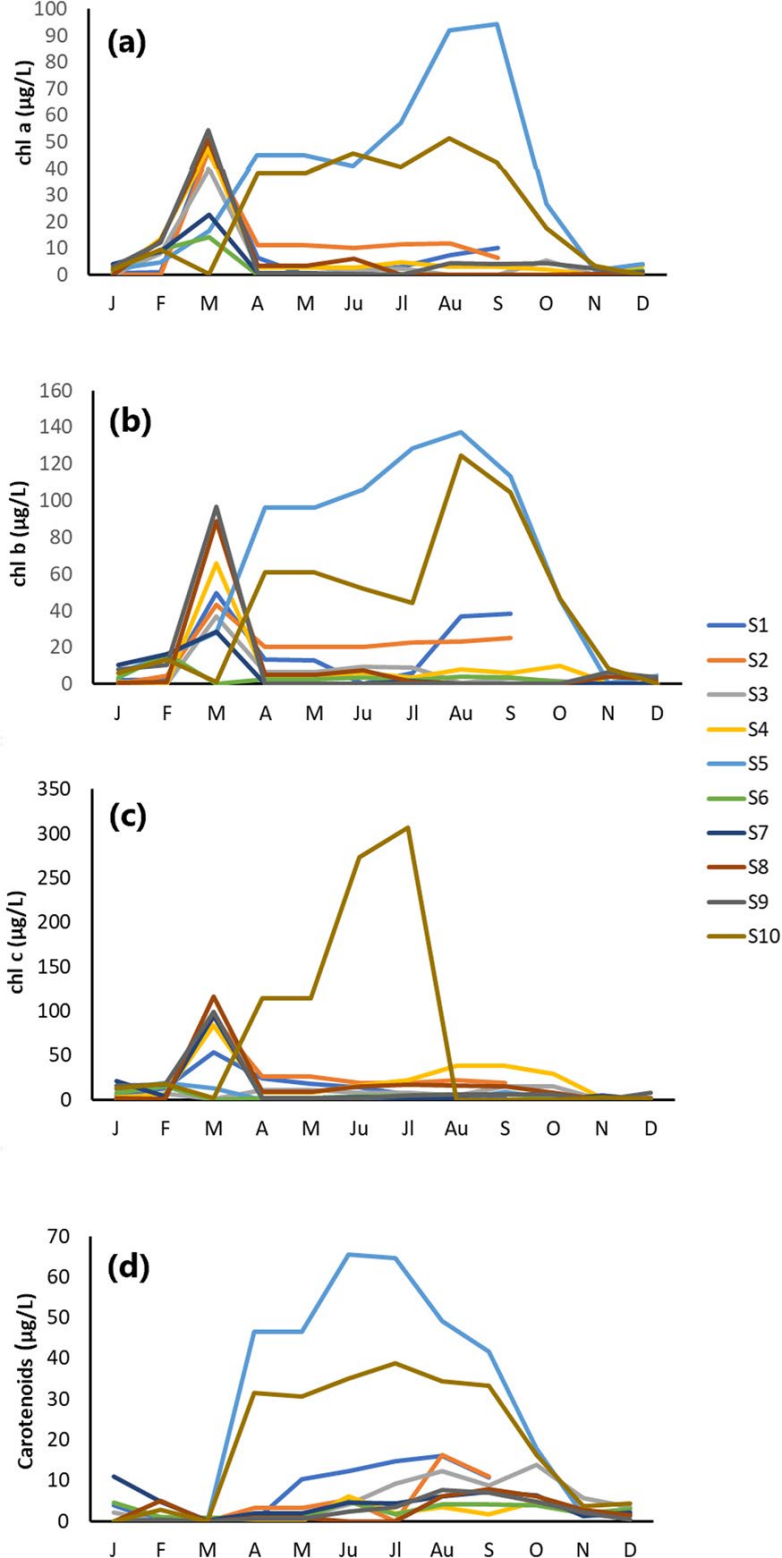
Monthly measurements of Almyros water photosynthetic pigments: **a** chl a, **b** chl b, **c** chl c and **d** carotenoids of samples from January 2022 to December 2022 at the ten sampling stations

Figure 7 shows photosynthetic pigments’ monthly and spatial distributions, while Table 2 presents the minimum and maximum values and the mean. Chlorophyll-a concentrations fluctuated during the sampling period (Fig. 7a), with an increase at stations 5 (from 1.57 to 94.27 μg/L) and 10 (from 0 to 51.38 μg/L). Especially in March, chlorophyll-a levels were elevated at all stations except for station 10 (Fig. 7a). The maximum chlorophyll-a values were observed at stations 5 and 10 from April to September. Chlorophyll-a at station 5 was 91.93 μg/L in August and 94.27 μg/L in September (Fig. 7a).

Chlorophyll-b values (Fig. 7b) followed a similar trend to chlorophyll-a, ranging from 0 to 137.2 μg/L (at station 5 in August). Chlorophyll-b was elevated at stations 5 and 10 from April to September, except station 10 in July (Fig. 7b). A decrease in chlorophyll-b was observed at stations 6 and 10 in March, in contrast to all other stations, where an increase was observed (Fig. 7b).

The distribution of chlorophyll-c (Fig. 7c) was slightly different to the other chlorophylls and ranged from 0 to 307.12 μg/L (at station 10 in July), which is also the highest value of chlorophyll-c. At station 10, a significant increase in chlorophyll-c was observed in June (273.06 μg/L) and July (307.12 μg/L), in contrast to chlorophyll-a and chlorophyll-b in the same month. This may be due to the proximity to the sea, where marine algae like diatoms and brown algae usually dominate. In March, chlorophyll-c decreased at stations 3, 5, 6 and 10 and increased at the other stations in the same month (Fig. 7b).

From April to August, when the temperature increases, chlorophyll-a and chlorophyll-b start to increase at stations 5 and 10 (Fig. 7a, b). In addition, chlorophyll-a and chlorophyll-b decreased at stations 6 and 10 in March.

Carotenoids ranged from 0 to 65.47 μg/L and increased steadily from spring to autumn, especially at stations 5 and 10, while they decreased in winter (Fig. 7d).

The photosynthetic pigments chlorophyll-a, chlorophyll-b, chlorophyll-c and carotenoids fluctuated more at stations 5 and 10, while the minimum values were observed in the cold months (November, December and January) (Fig. 7a–d). Stations 5 and 10 are in places with low water flow. Chlorophyll-b and chlorophyll-c occurred in greater amounts than chlorophyll-a.

#### Correlations

Statistical processing regarding the correlation among the physicochemical parameters, nutrients and pigments, provided: (1) a strong correlation of 0.75 between carotenoids and chl a (Fig. 11a, Appendix I), (2) an even stronger correlation of 0.85 between carotenoids and chl b (Fig. 11b, Appendix I) and (3) a moderate correlation between nitrate and pH (Fig. 11c, Appendix I).

### Soil characterisation

**Fig. 8:**
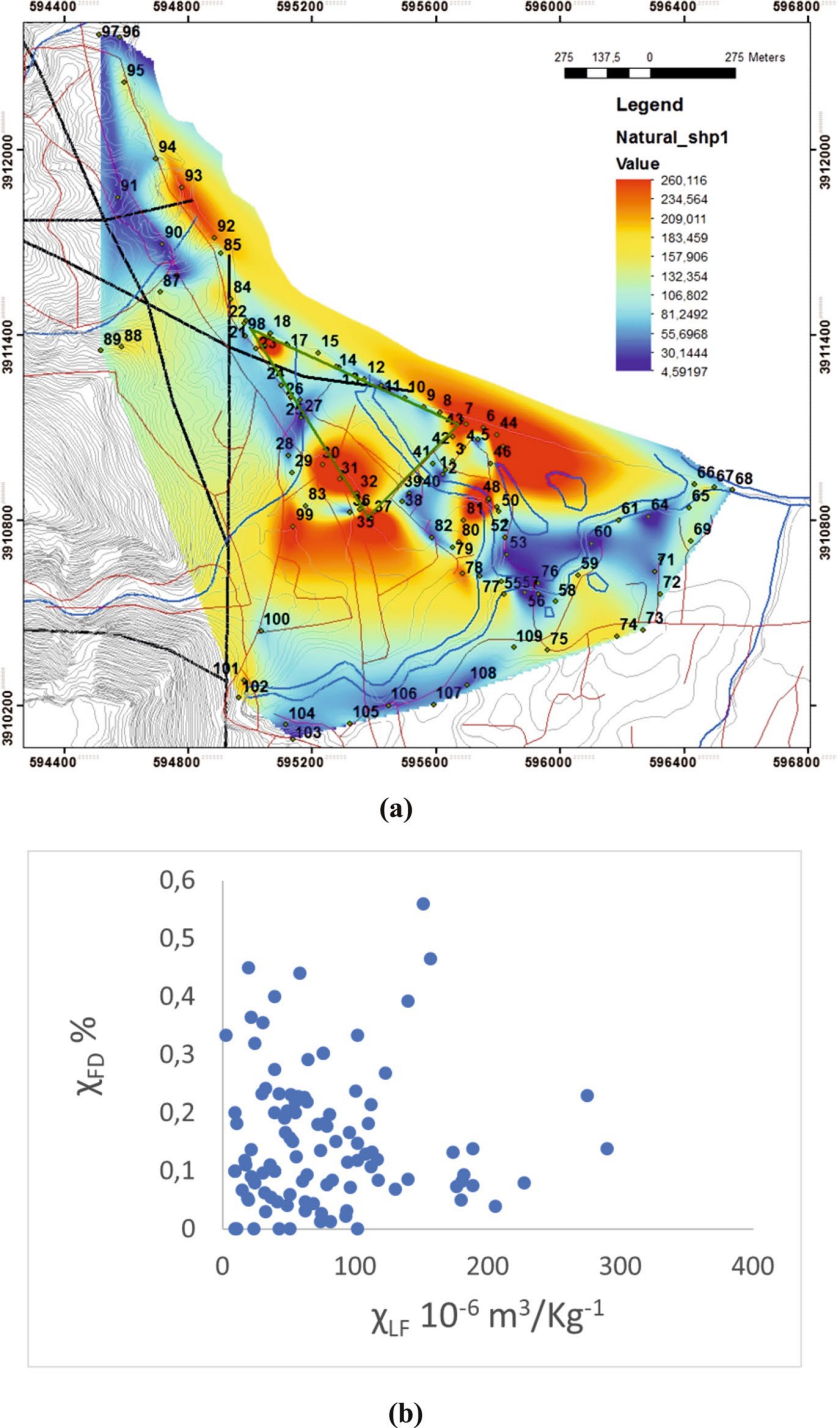
Spatial distribution of magnetic susceptibility **a** low-field magnetic susceptibility (LFS), **b** frequency-dependent susceptibility χ% (Kokinou et al., 2013). Black line - fault, blue line - drainage network, green line - power plant, red line - transport network, black circle - sample location

Figure 8a shows the spatial distribution of low-frequency magnetic susceptibility (χ_LF_). High χ_LF_ values, indicating potentially contaminated sites, are generally found near the power plant (Figs. 1B, and Fig. 8a), the transport network and the coast. Most samples selected throughout the Almyros network have relatively low χ_LF_ values.

Figure 8b shows the plot of frequency-dependent susceptibility (χ_FD_) versus low-frequency magnetic susceptibility (χ_LF_). According to Dearing et al. (1996) if χ_FD_ fraction is less than 2%, and χ_LF_ is greater than 0.5 10^−6^ m^3^/Kg^−1^, the samples are mostly pollution particles and igneous rocks containing single-domain and multidomain ferrimagnetic grains.

**Fig. 9:**
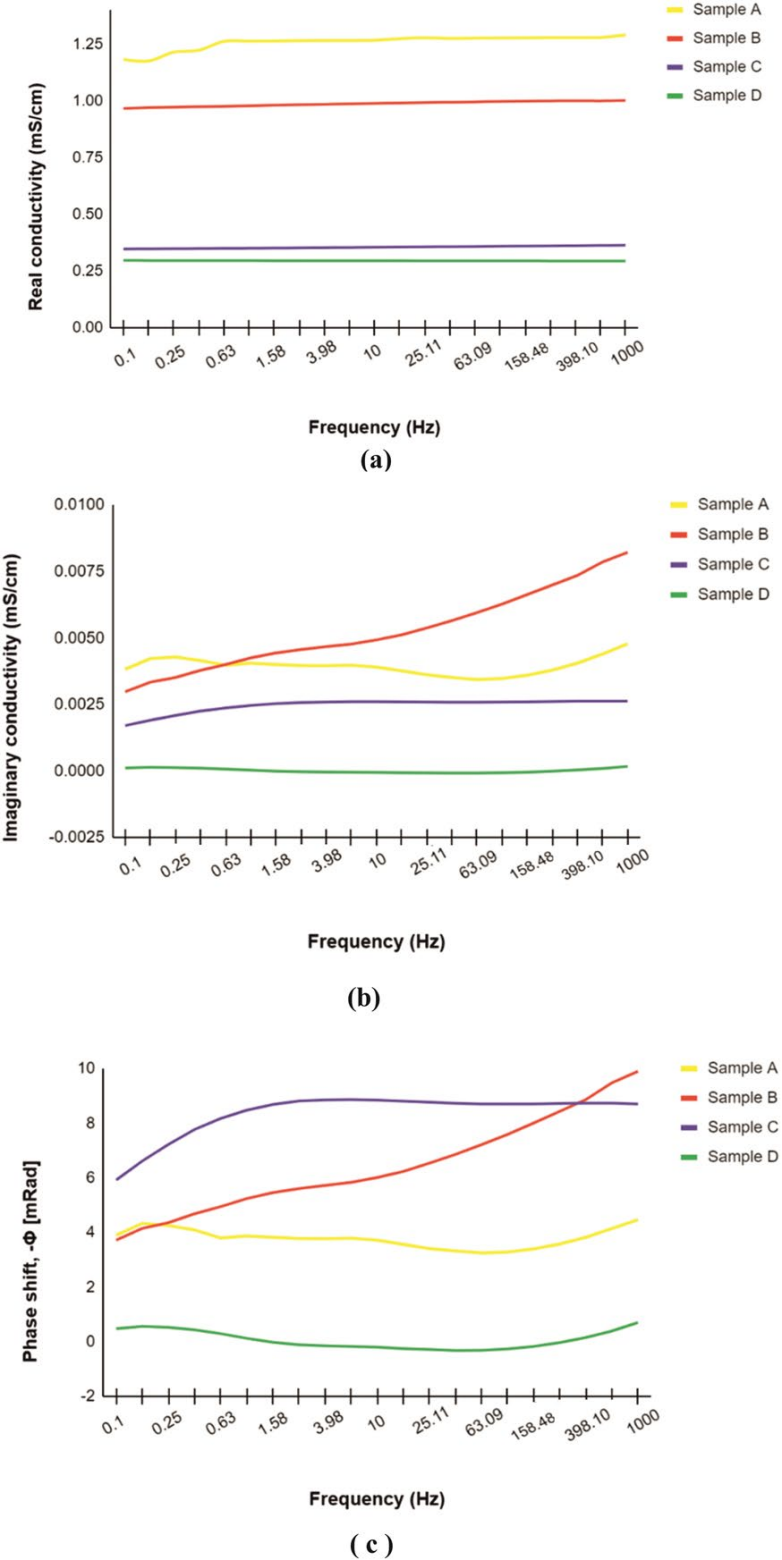
SIP response of Almyros soil saturated with tap water: **a** and **b** real and imaginary conductivity versus frequency based on geophysical measurements (spectral induced polarization) corresponding to May 2022 at the four sampling stations (location in Fig. 1), **c** frequency versus phase at the four sampling stations

**Fig. 10:**
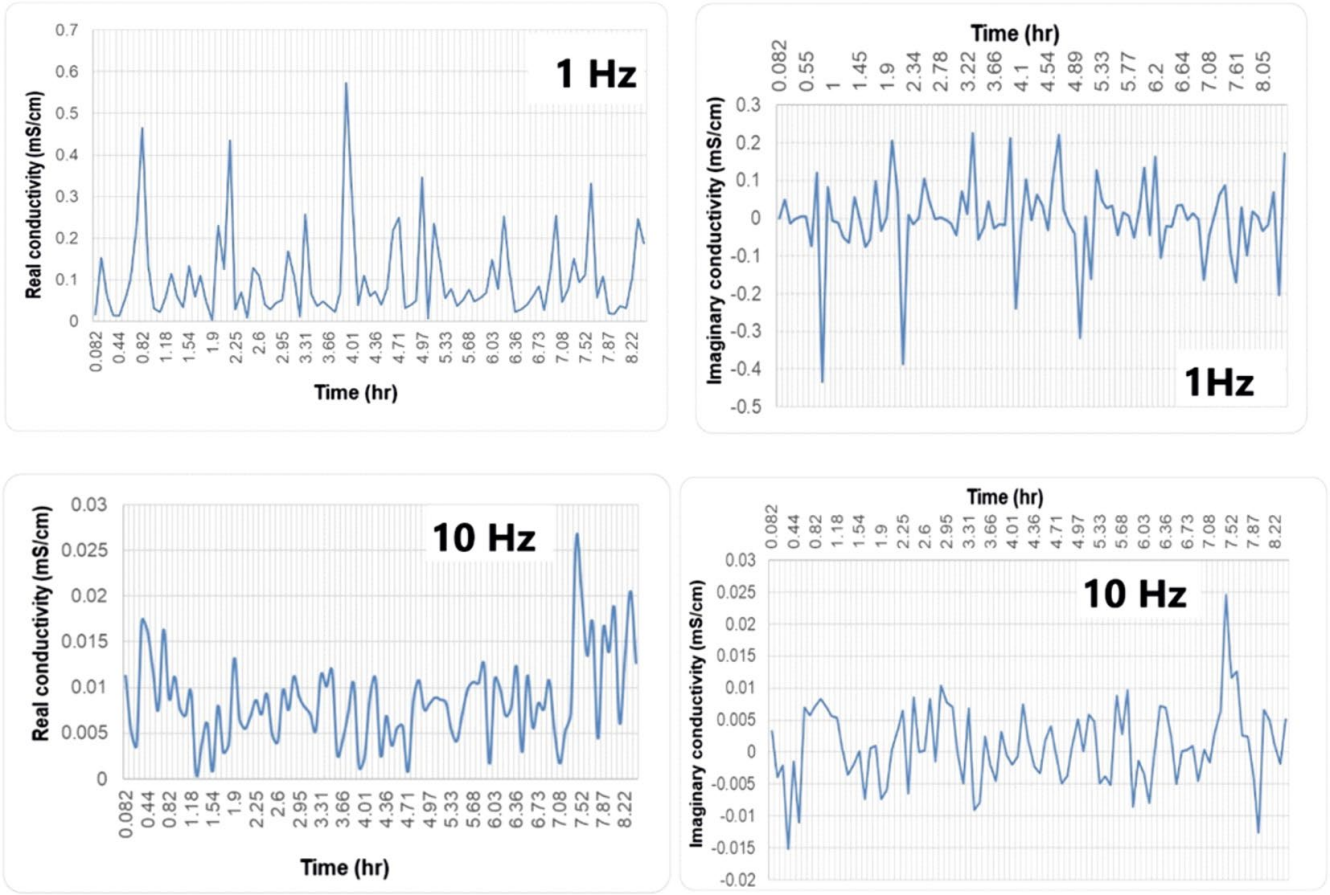
Soil from Almyros Wetland contaminated with oil: variation of SIP response (real conductivity and imaginary conductivity) at 1Hz (upper part) and 10 Hz (lower part) with time

The SIP response of the Almyros soil saturated with water is shown in Fig. 9a–c. The real conductivity of the four samples (A, B, C, D) ranges from 0.3 to 1.29 mS/cm in the frequency range 0-1000 Hz (Fig. 9a). The imaginary conductivity shows values between −8 × 10^−5^ and 0.005 mS/cm (Fig. 9b), while the phase shows slight variations (0–9 mRad) with increasing frequency for all samples (Fig. 9c). Figure 10 shows the distribution of real and imaginary conductivity of the oil-contaminated soil at 1 Hz and 10 Hz frequencies during an 8-h experiment. The real conductivity of the oil-contaminated soil shows a mean of 0.104 mS/cm at 1 Hz and 0.008 mS/cm at 10 Hz during the 8-h experiment (Fig. 10, left part). The mean values of the real conductivity for the oil-saturated soil are lower compared to those of the water-saturated soil at the same frequencies (Figs. 9a and 10, left part). The same trend is observed for the imaginary conductivity. The mean value of the imaginary conductivity is −0.00295 mS/cm at 1 Hz, and 0.000954 mS/cm at 10 Hz (Fig. 10, right part) and is thus lower than the corresponding values of the water-saturated soil (Fig. 9b). Finally, the SIP response for water-saturated and oil-contaminated soils could serve as a reference for future environmental studies in Almyros wetland since this geophysical method is applied for the first time in this area.

## Discussion

### Spatiotemporal monitoring of water and soil in Almyros Karstic Wetland

Water conductivity, pH and dissolved oxygen are among the critical physical properties that, together with nutrient content, provide the chemistry of water for further construction of the wetland hydro-ecological conceptual model (Finlayson et al., 2018).

In the present study, water conductivity fluctuates both in different years (Fig. 4) and during the same year (Fig. 5b). Meteorological data for 2016 and 2022 show that 2016 was drier than 2022 (https://www.meteoblue.com/en/weather/). This could explain the differences in water conductivity (Figs. 4 and 5b). In addition, the values of the real conductivity (SIP geophysical response) of Almyros water (Fig. 5a) are close to the values obtained with the multiparameter probe for the same stations in May–June 2022 (Fig. 5b). The imaginary conductivity of SIP response shows large fluctuations in the frequency range 0–1000 Hz (Fig. 5c), indicating strong polarization effects (Binley & Kemna, 2005; Kemna et al., 2012; Slater & Lesmes, 2002). The water polarization could be due to the increased concentrations of photosynthetic pigments or nutrients (Fig. 7a–d). Considering the physicochemical parameters (Fig. 3, Table 2), the water body of the Almyros stream can be described as hard and very hard from January to March, except for station 3, where the water is very hard in all months, similar to seawater. Concerning pH, the water body is slightly alkaline because the mean value was 8.08.

Generally, ammonium in natural waters is present in very low amounts due to the degradation of nitrogen compounds by microbes (Maksimović et al., 2020). Ammonium levels higher than 0.1 mg/L, as in the present case (Fig. 6a, Table 2), are possibly due to the leaching of N from farm manure and/or pollution from sewage (Maksimović et al., 2020). The nitrate increase in winter (Fig. 6b) could be due to low consumption by phytoplankton (Long et al., 1995) or agricultural soil leaching during floods. In addition, the higher nitrate concentrations in winter could be due to the low consumption of phytoplankton (Macdonald & Hoffman, 1995). Orthophosphate followed the same changes in all months, with the highest values in September and the lowest in April, July and August (Fig. 7c). This could be due to the increase in phytoplankton in spring, leading to a decrease in phosphorate. Silica concentrations (Fig. 6d) in winter (February, October and November) were higher than in summer. Dissolved Si levels were lower in summer, probably due to algal growth. Both N and P were elevated in March, possibly due to agricultural and port activities (Denant et al., 1990). In addition, the decrease in N and P and the increase in pH in April show possible changes in photosynthetic and respiration processes (Hartmann et al., 2007).

Chlorophylls and carotenoids are essential biological compounds that are widely distributed in green plants. In the aquatic environment, the pigments can be degraded in response to chemical, photochemical and biological processes (Kuczynska et al., 2015). Chlorophylls depend on the phosphorus concentration in the water, which is necessary for algal growth (Mineeva, 2022), on the oxygen content, pH and nitrate (Dar et al., 2013; Horvatić et al., 2013). Specifically, these authors stated that the concentration of chlorophyll increases when the nutrients in the water are increased and that the amount of chlorophyll in the water is usually high in summer and low in winter, so monitoring chlorophyll at different times of the year is essential. Among the forms of chlorophyll, chlorophyll-a is perhaps the most critical biological indicator for assessing lake productivity and water quality. Together with phosphorus, chlorophyll is often used to estimate the productivity of a water body. Although there are no binding values for chlorophyll concentrations in water, specific trophic classification systems for lake waters have been proposed (OECD., 1982). Low productivity means lakes are called oligotrophic, and high productivity means lakes are called eutrophic (Farrell-Poe, 2002). The status of a water body is considered oligotrophic when the maximum chlorophyll-a content is below 8 μg/L, mesotrophic when the content is between 8 and 25 μg/L, eutrophic when the content is between 26 and 75 μg/L, and hypertrophic when the content is above 75 μg/L (OECD., 1982).

Chlorophylls and carotenoids (Fig. 7a–d) in the present study had high mean concentrations throughout the year (Fig. 7, Table 2), indicating high algal productivity (Tani et al., 2009). The stream can be classified as a eutrophic water body characterised by ultraplanktonic forms (DataStream, 2023; Wetzel, 1983) as chlorophyll-a levels are relatively high, especially at stations 5 and 10 as shown in Table 2. Chlorophyll-a and chlorophyll-b values changed during spring and summer at these stations (Table 2), possibly due to changes in plant growth, photosynthetic capacity, or the influence of various stress factors at these sites (Beck & Redman, 1940; Lukić et al., 2020). Chlorophyll-a and -b increase at the beginning of the summer and in autumn can be explained by the availability of nutrients (Kitajima & Hogan, 2003; Wetzel, 2001). The maximum increase in chlorophyll-a in summer and autumn and the phosphorus levels, which were highest in autumn, represent nutrient sinks for inorganic nutrients (Pingree et al., 1977), and the sudden decrease in chlorophyll-c in summer could be due to stress from high temperatures (Maksimović et al. 2020).

The correlations between chl a-carotenoids and the chl b-carotenoids for the dry period are strong (Fig. 11, Appendix I). This can be explained by the presence of chlorophyta and prochlorophyta as these algae consist of chl a, chl b and carotenoids. Furthermore, pH showed a moderate negative correlation with N-NO3 during the dry period. Such moderate correlations have been observed in coastal surface water (Gopinath et al., 2002) and groundwater samples (Saalidong et al., 2022). In Almyros Stream, this could be related to atmospheric deposition due to fossil fuel combustion in the power plant (Fig. 1B). It is worth noting that the correlations between photosynthetic pigments and nutrients (N-NO_3_, N-NH_4_, P-PO_4_) were not appreciable as usually indicated for other streams and aquatic systems (Dodds & Smith, 2016; Khungwayo, 2022). None of these nutrients is the limiting factor for algal growth in Almyros Stream. The limiting factor could be among other factors, such as flow rate, flow velocity and temperature, which were not investigated in this work.

Plots of soil frequency-dependent susceptibility (χ_FD_) versus low-frequency magnetic susceptibility (χ_LF_) show that the χ_FD_ fraction is less than 2% and χ_LF_ is greater than 0.5 10–6 m3/Kg^−1^ (Fig. 8b) according to Dearing et al. (1996). High values of magnetic susceptibility χLF are oriented towards NW-SE (Fig. 8a), which corresponds to the orientation of the study area, while low values of magnetic susceptibility χLF are distributed mainly in the south-eastern part of the study area. In the absence of igneous rocks in the study area, these samples are (a) largely dominated by frequency-independent grains and (b) most likely associated with pollution particles. The effects of pollution could be exacerbated by climate change. The wind is probably the most transmission factor of heavy metals polluted dust in the study area. The extensive, almost straight front of the coast is unprotected from the winds of the northern sector (mainly north, northeast and northwest) (Pehlivanoglou & Papathanasoglou, 2004).

## Conclusions

The present study investigates the application of chemical properties (physicochemical parameters, nutrients, photosynthetic pigments) supported by geophysical analysis (spectral-induced polarization, magnetic susceptibility) and ecological mapping integrated with GIS to characterise, both qualitatively and quantitatively, the karstic wetland of the Almyros Stream in Heraklion, Crete, Greece. This research led to the following results:

- The water conductivity of the Almyros Wetland shows large monthly and annual fluctuations and is closely linked to seasonal discharge inputs and yearly weather conditions. In addition, spectral induced polarization (real and imaginary components) can serve as an excellent, fast and inexpensive monitoring tool. In particular, imaginary conductivity in the present case indicates water polarization, possibly due to the increased concentrations of photosynthetic pigments or nutrients.

- The Almyros Stream did not show significant spatial variations in physicochemical parameters. Phosphate, nitrate and silicate showed no spatial but only monthly fluctuations. Ammonium concentration showed spatial and monthly fluctuations. The photosynthetic pigments (chlorophyll-a, chlorophyll-b, chlorophyll-c) and carotenoids fluctuated mainly at stations 5 and 10, while the minimum values were observed in the cold months (November, December and January). Considering the high levels of nutrients such as P and N and the high levels of photosynthetic pigments, the trophic type of the Almyros Stream is eutrophic and tends to become hypereutrophic. This is possibly related to agricultural and industrial activities. Chlorophyll-a and -b showed strong positive correlation with carotenoids for the dry period (April–October), possibly related to the presence of chlorophyta and prochlorophyte. Furthermore, pH showed a moderate negative correlation with N-NO_3_ during the dry period, already observed in other cases of coastal surface water.

- The spatial distribution of low-frequency magnetic susceptibility possibly indicates contaminated sites due to the presence of heavy metals. The possible contaminated sites are located near the power plant, the transport network and the coast, while the low-frequency magnetic susceptibility shows relatively low values along the Almyros network. Since no igneous rocks are in the study area, these samples are primarily dominated by frequency-independent grains and are most likely associated with pollution particles. The SIP response (real, imaginary and phase) of Almyros soil samples collected along the stream network and saturated with tap water shows no significant variations with frequency, possibly indicating non-polluted soils along stream, which is also confirmed by the distribution of magnetic susceptibility. In contrast, SIP shows significant variations for oil-polluted soils, confirming that SIP response could serve as a pollution indicator in the future.

- The combined application of chemical analysis (physicochemical parameters, nutrients, photosynthetic pigments), geophysical analysis (spectral induced polarization, magnetic susceptibility) and ecological mapping can provide an excellent monitoring system for karst wetlands.

## Supplementary information

ESM 1 (DOCX 164 kb)

## Data Availability

The datasets of the current study are available from the corresponding author on reasonable request.
