# Supplementary material for: Spatiotemporal environmental monitoring of the karst-related Almyros Wetland (Heraklion, Crete, Greece, Eastern Mediterranean)
Source: Environ Monit Assess. 2023 Jul 15;195(8):955. doi: 10.1007/s10661-023-11571-5 (PMC10349741; doi:10.1007/s10661-023-11571-5)
Supplement: Supplementary file 1 — (DOCX 164 kb) [file 10661_2023_11571_MOESM1_ESM.docx]

**Appendix I, Table 1** Plant species in Almyros Wetland

| **Plant Species** | **Family** |
| --- | --- |
| *Acacia retinodes* | Fabaceae |
| *Ammophila arenaria* | Poaceae |
| *Anagallis arvensis L.* | Primulaceae |
| *Apium graveolens* | Apiaceae |
| *Arum cylindraceum* | Araceae |
| *Arundo donax* | Poaceae |
| *Bromus racemosus* | Poaceae |
| *Calicotome villosa* | Fabaceae |
| *Carex pseudocyperus* | Cyperaceae |
| *Ceratonia siliqua* | Fabaceae |
| *Cota tinctoria* | Asteraceae |
| *Cynoglossum creticum* | Boraginaceae |
| *Drimia maritima* | Asparagaceae |
| *Eryngium maritimum* | Apiaceae |
| *Eucalyptus globulus* | Myrtaceae |
| *Euphorbia characias* | Euphorbiaceae |
| *Ficus carica* | Moraceae |
| *Foeniculum vulgare* | Apiaceae |
| *Glaucium flavum* | Papaveraceae |
| *Hyparrhenia hirta* | Poaceae |
| *Juncus spp.* | Juncaceae |
| *Leontodon tuberosus* | Asteraceae |
| *Ligustrum sp.* | Oleaceae |
| *Malva sylvestris* | Malvaceae |
| *Matricaria chamomilla* | Asteraceae |
| *Myriophyllum spicatum* | Haloragaceae |
| *Nicotiana glauca* | Solanaceae |
| *Olea europaea* | Oleaceae |
| *Olea oleaster* | Oleaceae |
| *Oxalis corniculata* | Oxalidaceae |
| *Pancratium maritimum* | Amaryllidaceae |
| *Phlomis fruticosa* | Lamiaceae |
| *Phoenix theophrasti* | Arecaceae |
| *Phragmites australis* | Poaceae |
| *Pinus silvestris* | Pinaceae |
| *Pyrus spinosa* | Rosaceae |
| *Rubus fruticosus* | Rosaceae |
| *Salicornia europaea* | Amaranthaceae |
| *Salvia sp.* | Lamiaceae |
| *Sarcopoterium spinosum* | Rosaceae |
| *Scirpus sp.* | Cyperaceae |
| *Silybum marianum* | Asteraceae |
| *Tamarix spp.* | Tamaricaceae |
| *Urtica dioica* | Urticaceae |
| *Verbascum spp.* | Scrophulariaceae |
| *Vitex agnus-castus* | Lamiaceae |


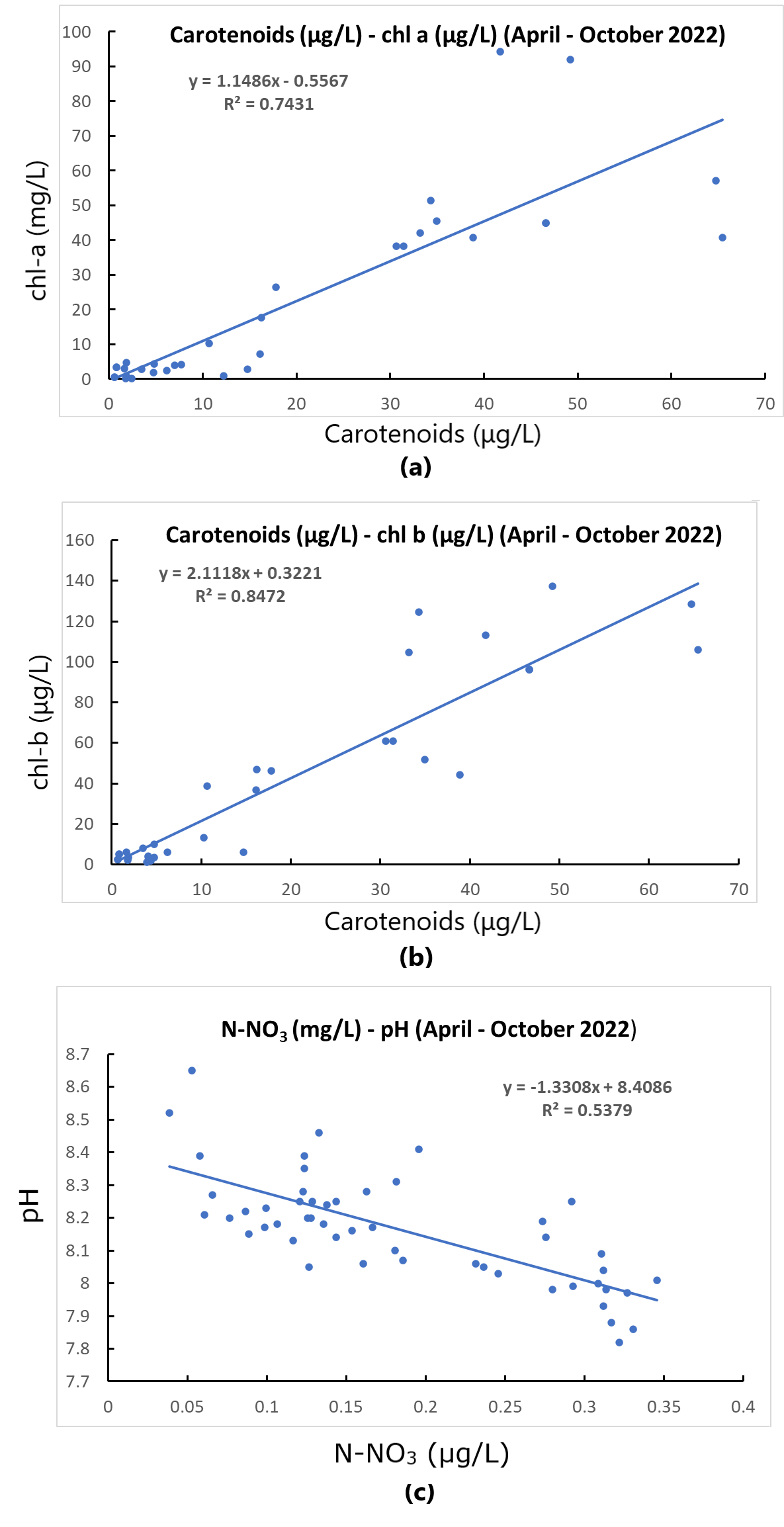


**Fig. 11** Correlation for the dry period (April-October 2022) between (a) carotenoids and chl-a, (b) carotenoids and chl-b and (c) N-NO_3_ and pH
